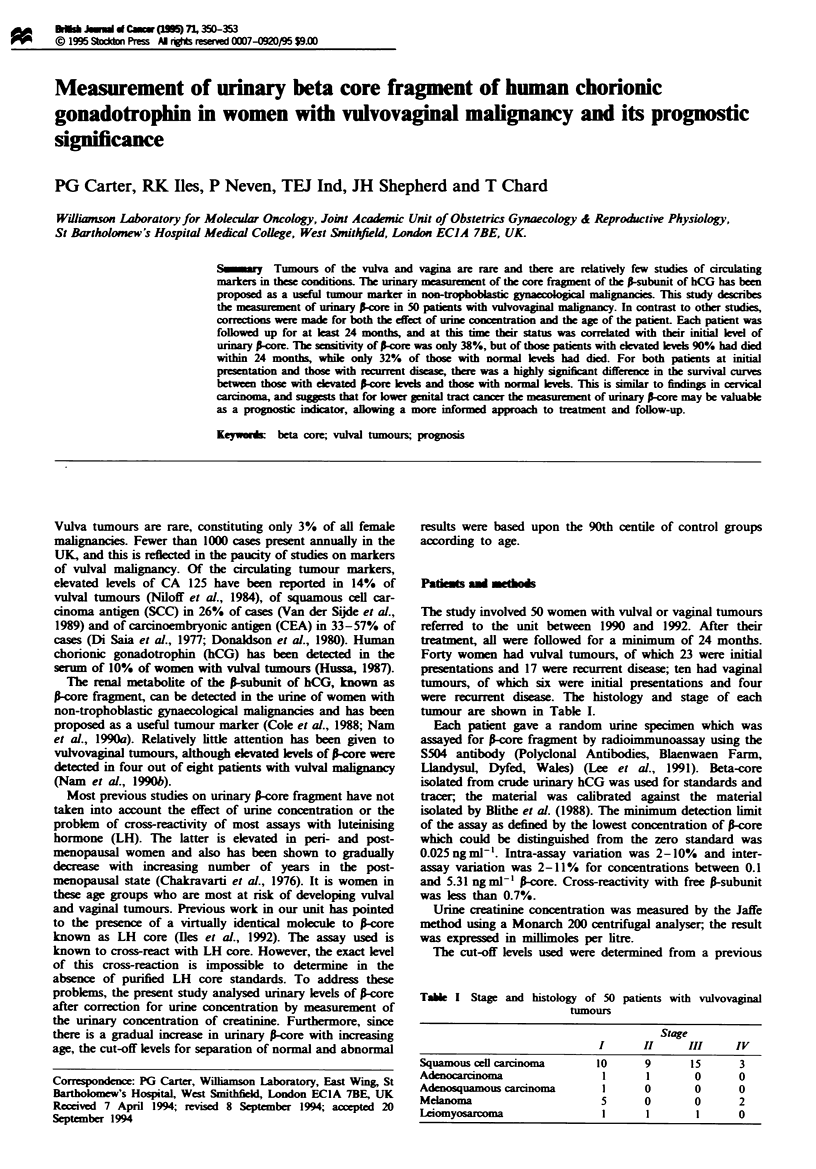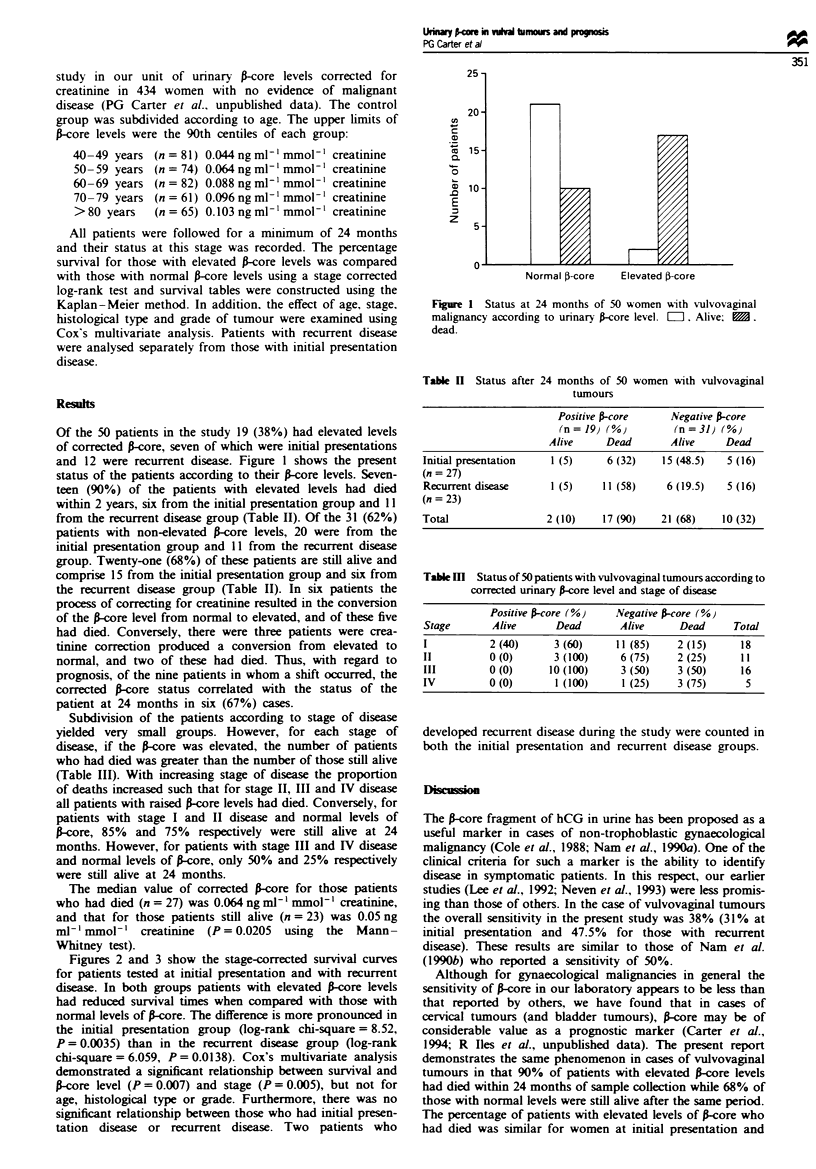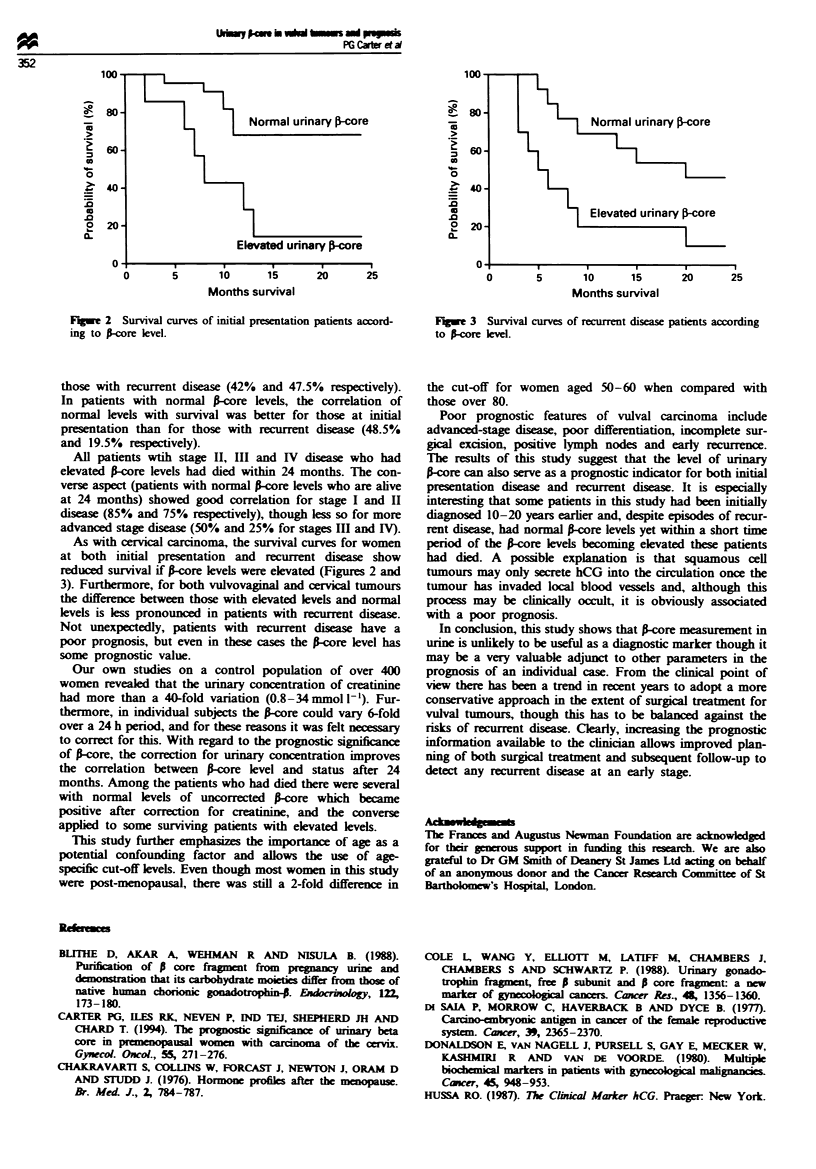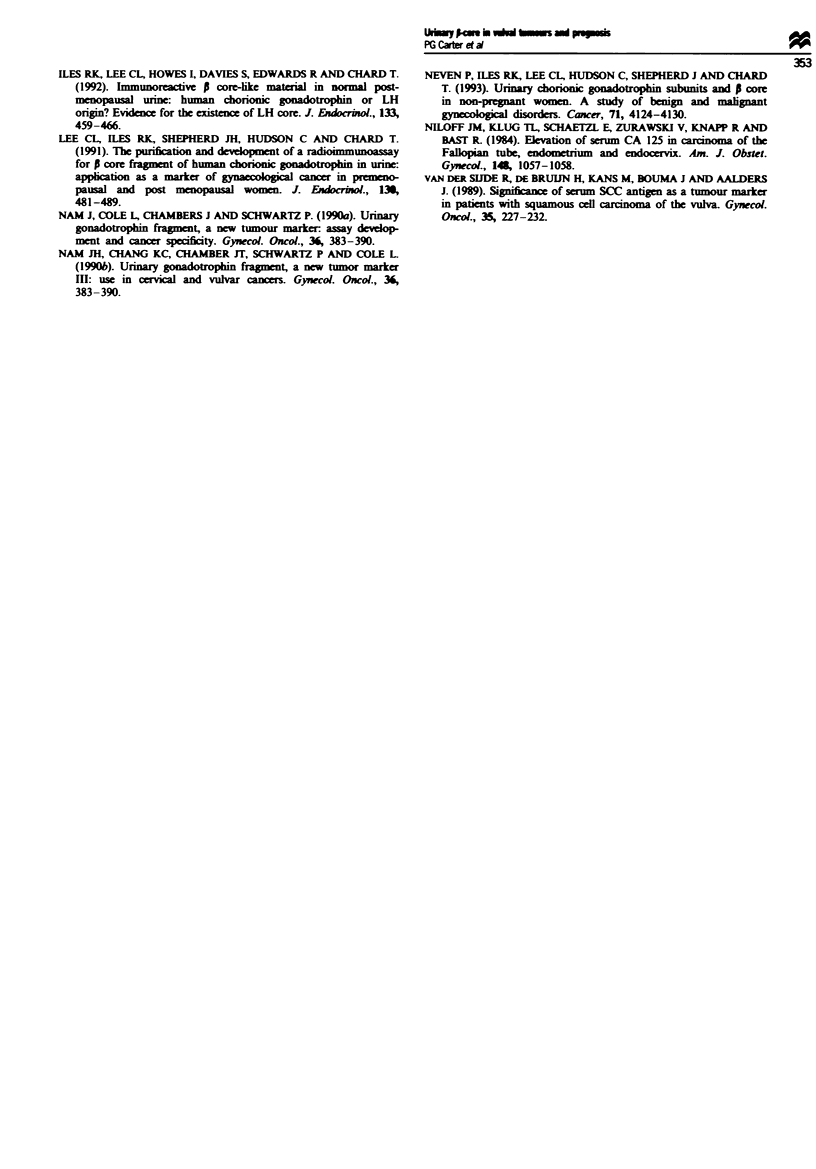# Measurement of urinary beta core fragment of human chorionic gonadotrophin in women with vulvovaginal malignancy and its prognostic significance.

**DOI:** 10.1038/bjc.1995.70

**Published:** 1995-02

**Authors:** P. G. Carter, R. K. Iles, P. Neven, T. E. Ind, J. H. Shepherd, T. Chard

**Affiliations:** Williamson Laboratory for Molecular Oncology, St Bartholomew's Hospital Medical College, West Smithfield, London, UK.

## Abstract

Tumours of the vulva and vagina are rare and there are relatively few studies of circulating markers in these conditions. The urinary measurement of the core fragment of the beta-subunit of hCG has been proposed as a useful tumour marker in non-trophoblastic gynaecological malignancies. This study describe the measurement of urinary beta-core in 50 patients with vulvovaginal malignancy. In contrast to other studies corrections were made for both the effect of urine concentration and the age of the patient. Each patient was followed up for at least 24 months, and at this time their status was correlated with their initial level of urinary beta-core. The sensitivity of beta-core was only 38%, but of those patients with elevated levels 90% had died within 24 months, while only 32% of those with normal levels had died. For both patients at initial presentation and those with recurrent disease, there was a highly significant difference in the survival curve between those with elevated beta-core levels and those with normal levels. This is similar to findings in cervical carcinoma, and suggests that for lower genital tract cancer the measurement of urinary beta-core may be valuable as a prognostic indicator, allowing a more informed approach to treatment and follow-up.


© 1995 Stockton Press All rights reserved 0007-0920/95 $9.00

### Measurement of urinary beta core fragment of human chorionic gonadotrophin in women with vulvovaginal malignancy and its prognostic significance

PG Carter, RK Iles, P Neven, TEJ Ind, JH Shepherd and T Chard

*Williamson Laboratory for Molecular Oncology, Joint Academic Unit of Obstetrics Gynaecology & Reproductive Physiology, St Bartholomew's Hospital Medical College, West Smithfield, London EC1A 7BE, UK.*

**Summary** Tumours of the vulva and vagina are rare and there are relatively few studies of circulating markers in these conditions. The urinary measurement of the core fragment of the β-subunit of hCG has been proposed as a useful tumour marker in non-trophoblastic gynaecological malignancies. This study describes the measurement of urinary β-core in 50 patients with vulvovaginal malignancy. In contrast to other studies, corrections were made for both the effect of urine concentration and the age of the patient. Each patient was followed up for at least 24 months, and at this time their status was correlated with their initial level of urinary β-core. The sensitivity of β-core was only 38%, but of those patients with elevated levels 90% had died within 24 months, while only 32% of those with normal levels had died. For both patients at initial presentation and those with recurrent disease, there was a highly significant difference in the survival curves between those with elevated β-core levels and those with normal levels. This is similar to findings in cervical carcinoma, and suggests that for lower genital tract cancer the measurement of urinary β-core may be valuable as a prognostic indicator, allowing a more informed approach to treatment and follow-up.

**Keywords:** beta core; vulval tumours; prognosis

Vulva tumours are rare, constituting only 3% of all female malignancies. Fewer than 1000 cases present annually in the UK, and this is reflected in the paucity of studies on markers of vulval malignancy. Of the circulating tumour markers, elevated levels of CA 125 have been reported in 14% of vulval tumours (Niloff *et al.*, 1984), of squamous cell carcinoma antigen (SCC) in 26% of cases (Van der Sijde *et al.*, 1989) and of carcinoembryonic antigen (CEA) in 33-57% of cases (Di Saia *et al.*, 1977; Donaldson *et al.*, 1980). Human chorionic gonadotrophin (hCG) has been detected in the serum of 10% of women with vulval tumours (Hussa, 1987).

The renal metabolite of the β-subunit of hCG, known as β-core fragment, can be detected in the urine of women with non-trophoblastic gynaecological malignancies and has been proposed as a useful tumour marker (Cole *et al.*, 1988; Nam *et al.*, 1990*a*). Relatively little attention has been given to vulvovaginal tumours, although elevated levels of β-core were detected in four out of eight patients with vulval malignancy (Nam *et al.*, 1990*b*).

Most previous studies on urinary β-core fragment have not taken into account the effect of urine concentration or the problem of cross-reactivity of most assays with luteinising hormone (LH). The latter is elevated in peri- and post-menopausal women and also has been shown to gradually decrease with increasing number of years in the post-menopausal state (Chakravarti *et al.*, 1976). It is women in these age groups who are most at risk of developing vulval and vaginal tumours. Previous work in our unit has pointed to the presence of a virtually identical molecule to β-core known as LH core (Iles *et al.*, 1992). The assay used is known to cross-react with LH core. However, the exact level of this cross-reaction is impossible to determine in the absence of purified LH core standards. To address these problems, the present study analysed urinary levels of β-core after correction for urine concentration by measurement of the urinary concentration of creatinine. Furthermore, since there is a gradual increase in urinary β-core with increasing age, the cut-off levels for separation of normal and abnormal results were based upon the 90th centile of control groups according to age.

Correspondence: PG Carter, Williamson Laboratory, East Wing, St Bartholomew's Hospital, West Smithfield, London EC1A 7BE, UK
Received 7 April 1994; revised 8 September 1994; accepted 20 September 1994

### Patients and methods

The study involved 50 women with vulval or vaginal tumours referred to the unit between 1990 and 1992. After their treatment, all were followed for a minimum of 24 months. Forty women had vulval tumours, of which 23 were initial presentations and 17 were recurrent disease; ten had vaginal tumours, of which six were initial presentations and four were recurrent disease. The histology and stage of each tumour are shown in Table I.

Each patient gave a random urine specimen which was assayed for β-core fragment by radioimmunoassay using the S504 antibody (Polyclonal Antibodies, Blaenwaen Farm, Llandysul, Dyfed, Wales) (Lee *et al.*, 1991). Beta-core isolated from crude urinary hCG was used for standards and tracer; the material was calibrated against the material isolated by Blithe *et al.* (1988). The minimum detection limit of the assay as defined by the lowest concentration of β-core which could be distinguished from the zero standard was $0.025\ ng\ ml^{-1}$. Intra-assay variation was 2-10% and inter-assay variation was 2-11% for concentrations between 0.1 and $5.31\ ng\ ml^{-1}$ β-core. Cross-reactivity with free β-subunit was less than 0.7%.

Urine creatinine concentration was measured by the Jaffe method using a Monarch 200 centrifugal analyser; the result was expressed in millimoles per litre.

The cut-off levels used were determined from a previous

**Table I** Stage and histology of 50 patients with vulvovaginal tumours

| | Stage | | | |
|---|---|---|---|---|
| | *I* | *II* | *III* | *IV* |
| Squamous cell carcinoma | 10 | 9 | 15 | 3 |
| Adenocarcinoma | 1 | 1 | 0 | 0 |
| Adenosquamous carcinoma | 1 | 0 | 0 | 0 |
| Melanoma | 5 | 0 | 0 | 2 |
| Leiomyosarcoma | 1 | 1 | 1 | 0 |

study in our unit of urinary β-core levels corrected for creatinine in 434 women with no evidence of malignant disease (PG Carter *et al.*, unpublished data). The control group was subdivided according to age. The upper limits of β-core levels were the 90th centiles of each group:

40–49 years ($n = 81$) 0.044 ng $ml^{-1}$ $mmol^{-1}$ creatinine

50–59 years ($n = 74$) 0.064 ng $ml^{-1}$ $mmol^{-1}$ creatinine

60–69 years ($n = 82$) 0.088 ng $ml^{-1}$ $mmol^{-1}$ creatinine

70–79 years ($n = 61$) 0.096 ng $ml^{-1}$ $mmol^{-1}$ creatinine

>80 years ($n = 65$) 0.103 ng $ml^{-1}$ $mmol^{-1}$ creatinine

All patients were followed for a minimum of 24 months and their status at this stage was recorded. The percentage survival for those with elevated β-core levels was compared with those with normal β-core levels using a stage corrected log-rank test and survival tables were constructed using the Kaplan–Meier method. In addition, the effect of age, stage, histological type and grade of tumour were examined using Cox's multivariate analysis. Patients with recurrent disease were analysed separately from those with initial presentation disease.

### Results

Of the 50 patients in the study 19 (38%) had elevated levels of corrected β-core, seven of which were initial presentations and 12 were recurrent disease. Figure 1 shows the present status of the patients according to their β-core levels. Seventeen (90%) of the patients with elevated levels had died within 2 years, six from the initial presentation group and 11 from the recurrent disease group (Table II). Of the 31 (62%) patients with non-elevated β-core levels, 20 were from the initial presentation group and 11 from the recurrent disease group. Twenty-one (68%) of these patients are still alive and comprise 15 from the initial presentation group and six from the recurrent disease group (Table II). In six patients the process of correcting for creatinine resulted in the conversion of the β-core level from normal to elevated, and of these five had died. Conversely, there were three patients were creatinine correction produced a conversion from elevated to normal, and two of these had died. Thus, with regard to prognosis, of the nine patients in whom a shift occurred, the corrected β-core status correlated with the status of the patient at 24 months in six (67%) cases.

Subdivision of the patients according to stage of disease yielded very small groups. However, for each stage of disease, if the β-core was elevated, the number of patients who had died was greater than the number of those still alive (Table III). With increasing stage of disease the proportion of deaths increased such that for stage II, III and IV disease all patients with raised β-core levels had died. Conversely, for patients with stage I and II disease and normal levels of β-core, 85% and 75% respectively were still alive at 24 months. However, for patients with stage III and IV disease and normal levels of β-core, only 50% and 25% respectively were still alive at 24 months.

The median value of corrected β-core for those patients who had died ($n = 27$) was 0.064 ng $ml^{-1}$ $mmol^{-1}$ creatinine, and that for those patients still alive ($n = 23$) was 0.05 ng $ml^{-1}$ $mmol^{-1}$ creatinine ($P = 0.0205$ using the Mann–Whitney test).

Figures 2 and 3 show the stage-corrected survival curves for patients tested at initial presentation and with recurrent disease. In both groups patients with elevated β-core levels had reduced survival times when compared with those with normal levels of β-core. The difference is more pronounced in the initial presentation group (log-rank chi-square = 8.52, $P = 0.0035$) than in the recurrent disease group (log-rank chi-square = 6.059, $P = 0.0138$). Cox's multivariate analysis demonstrated a significant relationship between survival and β-core level ($P = 0.007$) and stage ($P = 0.005$), but not for age, histological type or grade. Furthermore, there was no significant relationship between those who had initial presentation disease or recurrent disease. Two patients who developed recurrent disease during the study were counted in both the initial presentation and recurrent disease groups.

**Figure 1** Status at 24 months of 50 women with vulvovaginal malignancy according to urinary β-core level. □, Alive; ▨, dead.

**Table II** Status after 24 months of 50 women with vulvovaginal tumours

| | Positive β-core (n = 19) (%) Alive | Dead | Negative β-core (n = 31) (%) Alive | Dead |
|---|---|---|---|---|
| Initial presentation ($n = 27$) | 1 (5) | 6 (32) | 15 (48.5) | 5 (16) |
| Recurrent disease ($n = 23$) | 1 (5) | 11 (58) | 6 (19.5) | 5 (16) |
| Total | 2 (10) | 17 (90) | 21 (68) | 10 (32) |

**Table III** Status of 50 patients with vulvovaginal tumours according to corrected urinary β-core level and stage of disease

| Stage | Positive β-core (%) Alive | Dead | Negative β-core (%) Alive | Dead | Total |
|---|---|---|---|---|---|
| I | 2 (40) | 3 (60) | 11 (85) | 2 (15) | 18 |
| II | 0 (0) | 3 (100) | 6 (75) | 2 (25) | 11 |
| III | 0 (0) | 10 (100) | 3 (50) | 3 (50) | 16 |
| IV | 0 (0) | 1 (100) | 1 (25) | 3 (75) | 5 |

### Discussion

The β-core fragment of hCG in urine has been proposed as a useful marker in cases of non-trophoblastic gynaecological malignancy (Cole *et al.*, 1988; Nam *et al.*, 1990*a*). One of the clinical criteria for such a marker is the ability to identify disease in symptomatic patients. In this respect, our earlier studies (Lee *et al.*, 1992; Neven *et al.*, 1993) were less promising than those of others. In the case of vulvovaginal tumours the overall sensitivity in the present study was 38% (31% at initial presentation and 47.5% for those with recurrent disease). These results are similar to those of Nam *et al.* (1990*b*) who reported a sensitivity of 50%.

Although for gynaecological malignancies in general the sensitivity of β-core in our laboratory appears to be less than that reported by others, we have found that in cases of cervical tumours (and bladder tumours), β-core may be of considerable value as a prognostic marker (Carter *et al.*, 1994; R Iles *et al.*, unpublished data). The present report demonstrates the same phenomenon in cases of vulvovaginal tumours in that 90% of patients with elevated β-core levels had died within 24 months of sample collection while 68% of those with normal levels were still alive after the same period. The percentage of patients with elevated levels of β-core who had died was similar for women at initial presentation and

**Figure 2** Survival curves of initial presentation patients according to β-core level.

**Figure 3** Survival curves of recurrent disease patients according to β-core level.

those with recurrent disease (42% and 47.5% respectively). In patients with normal β-core levels, the correlation of normal levels with survival was better for those at initial presentation than for those with recurrent disease (48.5% and 19.5% respectively).

All patients wtih stage II, III and IV disease who had elevated β-core levels had died within 24 months. The converse aspect (patients with normal β-core levels who are alive at 24 months) showed good correlation for stage I and II disease (85% and 75% respectively), though less so for more advanced stage disease (50% and 25% for stages III and IV).

As with cervical carcinoma, the survival curves for women at both initial presentation and recurrent disease show reduced survival if β-core levels were elevated (Figures 2 and 3). Furthermore, for both vulvovaginal and cervical tumours the difference between those with elevated levels and normal levels is less pronounced in patients with recurrent disease. Not unexpectedly, patients with recurrent disease have a poor prognosis, but even in these cases the β-core level has some prognostic value.

Our own studies on a control population of over 400 women revealed that the urinary concentration of creatinine had more than a 40-fold variation (0.8–34 mmol $l^{-1}$). Furthermore, in individual subjects the β-core could vary 6-fold over a 24 h period, and for these reasons it was felt necessary to correct for this. With regard to the prognostic significance of β-core, the correction for urinary concentration improves the correlation between β-core level and status after 24 months. Among the patients who had died there were several with normal levels of uncorrected β-core which became positive after correction for creatinine, and the converse applied to some surviving patients with elevated levels.

This study further emphasizes the importance of age as a potential confounding factor and allows the use of age-specific cut-off levels. Even though most women in this study were post-menopausal, there was still a 2-fold difference in the cut-off for women aged 50–60 when compared with those over 80.

Poor prognostic features of vulval carcinoma include advanced-stage disease, poor differentiation, incomplete surgical excision, positive lymph nodes and early recurrence. The results of this study suggest that the level of urinary β-core can also serve as a prognostic indicator for both initial presentation disease and recurrent disease. It is especially interesting that some patients in this study had been initially diagnosed 10–20 years earlier and, despite episodes of recurrent disease, had normal β-core levels yet within a short time period of the β-core levels becoming elevated these patients had died. A possible explanation is that squamous cell tumours may only secrete hCG into the circulation once the tumour has invaded local blood vessels and, although this process may be clinically occult, it is obviously associated with a poor prognosis.

In conclusion, this study shows that β-core measurement in urine is unlikely to be useful as a diagnostic marker though it may be a very valuable adjunct to other parameters in the prognosis of an individual case. From the clinical point of view there has been a trend in recent years to adopt a more conservative approach in the extent of surgical treatment for vulval tumours, though this has to be balanced against the risks of recurrent disease. Clearly, increasing the prognostic information available to the clinician allows improved planning of both surgical treatment and subsequent follow-up to detect any recurrent disease at an early stage.

**Acknowledgements**

The Frances and Augustus Newman Foundation are acknowledged for their generous support in funding this research. We are also grateful to Dr GM Smith of Deanery St James Ltd acting on behalf of an anonymous donor and the Cancer Research Committee of St Bartholomew's Hospital, London.

**References**

BLITHE D, AKAR A, WEHMAN R AND NISULA B. (1988). Purification of β core fragment from pregnancy urine and demonstration that its carbohydrate moieties differ from those of native human chorionic gonadotrophin-β. *Endocrinology*, **122**, 173–180.

CARTER PG, ILES RK, NEVEN P, IND TEJ, SHEPHERD JH AND CHARD T. (1994). The prognostic significance of urinary beta core in premenopausal women with carcinoma of the cervix. *Gynecol. Oncol.*, **55**, 271–276.

CHAKRAVARTI S, COLLINS W, FORCAST J, NEWTON J, ORAM D AND STUDD J. (1976). Hormone profiles after the menopause. *Br. Med. J.*, **2**, 784–787.

COLE L, WANG Y, ELLIOTT M, LATIFF M, CHAMBERS J, CHAMBERS S AND SCHWARTZ P. (1988). Urinary gonadotrophin fragment, free β subunit and β core fragment: a new marker of gynecological cancers. *Cancer Res.*, **48**, 1356–1360.

DI SAIA P, MORROW C, HAVERBACK B AND DYCE B. (1977). Carcino-embryonic antigen in cancer of the female reproductive system. *Cancer*, **39**, 2365–2370.

DONALDSON E, VAN NAGELL J, PURSELL S, GAY E, MECKER W, KASHMIRI R AND VAN DE VOORDE. (1980). Multiple biochemical markers in patients with gynecological malignancies. *Cancer*, **45**, 948–953.

HUSSA RO. (1987). *The Clinical Marker hCG*. Praeger: New York.

ILES RK, LEE CL, HOWES I, DAVIES S, EDWARDS R AND CHARD T. (1992). Immunoreactive β core-like material in normal post-menopausal urine: human chorionic gonadotrophin or LH origin? Evidence for the existence of LH core. *J. Endocrinol.*, **133**, 459–466.

LEE CL, ILES RK, SHEPHERD JH, HUDSON C AND CHARD T. (1991). The purification and development of a radioimmunoassay for β core fragment of human chorionic gonadotrophin in urine: application as a marker of gynaecological cancer in premenopausal and post menopausal women. *J. Endocrinol.*, **130**, 481–489.

NAM J, COLE L, CHAMBERS J AND SCHWARTZ P. (1990*a*). Urinary gonadotrophin fragment, a new tumour marker: assay development and cancer specificity. *Gynecol. Oncol.*, **36**, 383–390.

NAM JH, CHANG KC, CHAMBER JT, SCHWARTZ P AND COLE L. (1990*b*). Urinary gonadotrophin fragment, a new tumor marker III: use in cervical and vulvar cancers. *Gynecol. Oncol.*, **36**, 383–390.

NEVEN P, ILES RK, LEE CL, HUDSON C, SHEPHERD J AND CHARD T. (1993). Urinary chorionic gonadotrophin subunits and β core in non-pregnant women. A study of benign and malignant gynecological disorders. *Cancer*, **71**, 4124–4130.

NILOFF JM, KLUG TL, SCHAETZL E, ZURAWSKI V, KNAPP R AND BAST R. (1984). Elevation of serum CA 125 in carcinoma of the Fallopian tube, endometrium and endocervix. *Am. J. Obstet. Gynecol.*, **148**, 1057–1058.

VAN DER SIJDE R, DE BRUIJN H, KANS M, BOUMA J AND AALDERS J. (1989). Significance of serum SCC antigen as a tumour marker in patients with squamous cell carcinoma of the vulva. *Gynecol. Oncol.*, **35**, 227–232.